# Complete mitochondrial genome of *Acropteris iphiata* (Lepidoptera: Uraniidae)

**DOI:** 10.1080/23802359.2019.1689865

**Published:** 2019-11-14

**Authors:** Ehsan Sanaei, Jeong Sun Park, Jun Seong Jeong, Min Jee Kim, Iksoo Kim

**Affiliations:** Department of Applied Biology, College of Agriculture & Life Sciences, Chonnam National University, Gwangju, Republic of Korea

**Keywords:** Mitochondrial genome, *Acropteris iphiata*, Uraniidae

## Abstract

*Acropteris iphiata* belongs to the family Uraniidae in the superfamily Geometroidea (Lepidoptera). We sequenced 15,346-bp long complete mitochondrial genome (mitogenome) of the species, which consists of a typical set of genes (13 protein-coding genes, 2 rRNA genes, and 22 tRNA genes) and one major non-coding A + T-rich region. The *A. iphiata* mitogenome harbored the gene order tRNA^Met^, tRNA^Ile^, and tRNA^Gln^ between the A + T-rich region and ND2 that is found in most lepidopteran mitogenomes. Bayesian inference (BI) and maximum likelihood (ML) phylogeny, using 13 protein-coding genes (PCGs) and 2 rRNAs showed that *A. iphiata* was placed as a sister to Geometridae with the highest nodal support (Bayesian posterior probabilities for BI = 1.00 and Bootstrap support for ML = 100).

*Acropteris iphiata* Achille Guenée, 1857, belongs to the family Uraniidae in the lepidopteran Geometroidea superfamily and is distributed in Korea, China, and Japan (Shin 2001). The larvae feed on *Metaplexis japonica* by cutting a leaf blade circularly between diverging veins (Nakamura and Yoshiyasu 1992). Final-instar larvae spin leaves, using a crude-meshed silken net, for pupation, which occurs in this species overwinter (Nakamura and Yoshiyasu 1992). Adults emerge from June to August (Shin 2001) and the wings of adults have several gray diagonal lines on a white background and a reddish-brown pattern on the tip of the front wing (Shin 2001; Heo 2012). Although Uraniidae consists of 90 genera, complete mitogenome sequences for this family have not yet been analyzed. Thus, mitogenome-based phylogeny for the superfamily Geometroidea is limited.

In order to sequence the complete mitogenome of *A. iphiata*, one adult was captured at Sinsong-ri, Heungdeok-myeon, Gochang-gun, Jeollabuk-do Province, South Korea (35°32′50.7″ N, 126°42′13.6″ E) and DNA was extracted from one hind leg. Leftover DNA and specimen were deposited at Chonnam National University, Gwangju, Korea, under the accession number, CNU 10888. Three overlapping long fragments (COI ∼ ND4 for LF1, ND5 ∼ lrRNA for LF2, and lrRNA ∼ COI for LF3) were amplified using three sets of Lepidoptera-specific primers (Kim et al. 2012). These LFs were used as templates for 26 short fragments. The other experimental methods used have been described in detail elsewhere (Kim et al. 2011, 2012).

The *A. iphiata* mitogenome is 15,346 bp in length, with typical gene sets (2 rRNAs, 22 tRNAs, and 13 PCGs) and a major non-coding A + T-rich region as 358 bp (GenBank accession number MN093120). The genome size of *A. iphiata* is well within the range found in Lepidoptera (Kim et al. 2010). The gene arrangement of *A. iphiata* is identical to the major lepidopteran type found in most lepidopteran species, which have the order tRNA^Met^–tRNA^Ile^–tRNA^Gln^ between the A + T-rich region and ND2 (Kim et al. 2010), instead of the ancestral type found in the majority of insects (Boore 1999).

Phylogenetic analysis using nucleotide sequences of 13 PCGs and two rRNA were conducted with ten species of Geometroidea, consisted of the families Geometridae and Uraniidae, to which *A. iphiata* belongs, and two outgroup species belonged to the Tortricoidea (Figure 1). Bayesian inference (BI) and maximum likelihood (ML) methods were conducted using MrBayes version 3.2.2 (Ronquist et al. 2012) and RAxML-HPC2 version 8.0.24 (Stamatakis 2014), respectively, which are incorporated in the CIPRES Portal version 3.1 (Miller et al. 2010). Ennominae and Larentiinae, which constitute the Geometridae, each represented a monophyletic group with the highest nodal supports, and Geometridae, as a monophyletic group, had high nodal support (Bayesian posterior probabilities by BI = 1.00 and Bootstrap support by ML = 98) (Figure 1). *Acropteris iphiata*, which belongs to the Uraniidae was placed as the sister group to Geometridae, forming the Geometroidea a monophyletic group with the highest nodal support (Figure 1). Currently, only *A. iphiata* mitogenome sequences are available for the family Uraniidae. Thus, additional mitogenome sequences from a diverse taxonomic group are required to infer the relationships among the families of Geometroidea.

**Figure 1.**
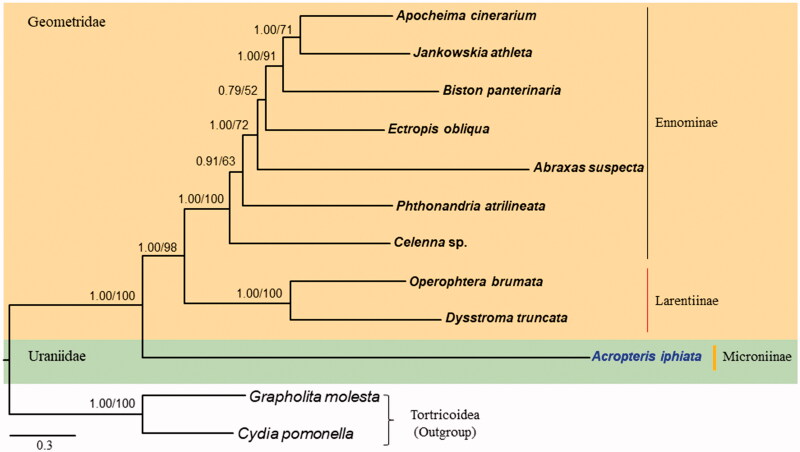
Phylogenetic tree for superfamily Geometroidea. The tree was constructed using nucleotide sequences of 13 protein-coding genes and two rRNAs via the Bayesian inference (BI) and Maximum Likelihood (ML) methods. The numbers at each node specify Bayesian posterior probabilities in percent by BI analysis and bootstrap percentages of 1000 pseudoreplicates by ML analysis. The scale bar indicates the number of substitutions per site. Two species of Tortricoidea (*Grapholita molesta* and *Cydia pomonella*) were included as outgroups. GenBank accession numbers are as follows: *Apocheima cinerarium*, KF836545 (Liu et al. 2014); *Jankowskia athleta*, KR822683 (Xu et al. 2016); *Biston panterinaria*, JX406146 (Yang et al. 2013); *Ectropis obliqua*, KX827002 (Unpublished); *Abraxas suspecta*, KY095828 (Sun et al. 2017); *Phthonandria atrilineata*, EU569764 (Yang et al. 2009); *Celenna* sp., KM244697 (Tang et al. 2014); *Operophtera brumata*, KP027400 (Derks et al. 2015); *Dysstroma truncata*, KJ508061 (Timmermans et al. 2014); *Grapholita molesta*, HQ116416 (Gong et al. 2012); and *Cydia pomonella*, JX407107 (Shi et al. 2013).
